# Differential aspects of stroke and congestive heart failure in quality of life reduction: a case series with three comparison groups

**DOI:** 10.1186/1477-7525-9-65

**Published:** 2011-08-10

**Authors:** Elen B Pinto, Iara Maso, Julio LB Pereira, Thiago G Fukuda, Jamile C Seixas, Daniela F Menezes, Carolina Cincura, Iuri S Neville, Pedro AP Jesus, Jamary Oliveira-Filho

**Affiliations:** 1 Stroke Clinic of the Federal University of Bahia, Ambulatório Magalhães Neto, Rua Padre Feijó 240 Canela, Bahia, Brazil; 2 Bahiana School of Medicine and Public Health Avenida Dom João VI, 274 - Brotas, Salvador, Bahia, Brazil

## Abstract

**Background:**

To assess QOL of patients with stroke in comparison to other groups (caregivers and CHF patients), to identify which items of QOL are more affected on each group and what is the functional profile of patients with stroke.

**Methods:**

Consecutive stroke or congestive heart failure (CHF) patients were evaluated and compared to their caregivers (caregivers). The NIH Stroke Scale (NIHSS) and EuroQoL-5D (EQ-5D) scale were applied.

**Results:**

We evaluated 67 patients with stroke, 62 with CHF and 67 caregivers. For stroke patients, median NIHSS score was four. EQ-5D score was significantly worse in stroke, as compared to CHF and caregivers (0.52, 0.69 and 0.65, respectively). Mobility and usual activity domains were significantly affected in stroke and CHF patients as compared to caregivers; and self-care was more affected in stroke as compared with the other two groups.

**Conclusions:**

Despite a mild neurological deficit, there was a significantly worse QOL perception in stroke as compared to CHF patients, mostly in their perception of self-care.

## Background

Stroke is one of the leading causes of death worldwide [1]. Two-thirds of stroke cases occur in developing countries, where prevalence is increasing as the population ages [2]. In Brazil, where stroke is the main cause of death, limited access to specialized stroke care and poor knowledge of risk factors and warning signs expose the population to a significant burden of disease [3]. Stroke survivors also impose a significant burden to society and caregivers. Another disease with significant burden to society is congestive heart failure (CHF). In Brazil, cardiac diseases represent the second most frequent cause of death [4]. While most heart diseases have experienced decreased morbidity and mortality over the past decades, CHF has remained stable and costs 46 billion dollars each year in the United States alone [5]. However, quantification of the impact of these diseases on other aspects of health care and morbidity in developing countries is lacking, such as functional outcome, activities of daily living and quality of life (QOL).

Several scales have been used to measure the impact of stroke and other diseases, most of which identify the perception of the health professional. Considerable emphasis has been given in recent years to the patient's perception of their own health process [6]. A significant proportion of patients considered independent by health professions have a significant impairment in QOL [7]. For example, patients with independent mobility may score well on a functional scale but have significant impairment in QOL due to unemployment or fear of disease worsening or recurrence.

In the present study, our objectives were: to measure QOL in patients with stroke, as compared to patients with CHF and caregivers (caregivers) and to correlate QOL with other known measures of stroke severity, such as the NIH Stroke Scale (NIHSS) and the modified Barthel Index (mBI).

## Methods

The study is a case series with three comparison groups (stroke, CHF and caregivers). Since age has a significant impact on QOL, the three groups were paired for age (aged within 5 years of the stroke group). Patients were selected between July, 2005 and November, 2007 from two subspecialty outpatient clinics (stroke and cardiomyopathy) from a university-based hospital in Salvador, Brazil. Stroke was defined by the presence of a focal neurological deficit of acute onset lasting over 24 hours, confirmed by neuroimaging (computed tomography of magnetic resonance imaging) and was established by the attending neurologist from the stroke clinic [8]. The diagnosis of CHF was based on signs and symptoms of low cardiac output and was established by the attending cardiologist from the cardiomyopathy clinic. In both populations, we excluded patients with osteo-articular causes of functional impairment. Caregivers were selected from both outpatient clinics. A standardized questionnaire was given to the caregiver population to exclude the following disease states: hypertension, diabetes, coronary heart disease, Chagas disease, depression, cancer, migraine, adult immunodeficiency syndrome, respiratory and osteo-articular diseases. Exclusion of these diseases was based on each individual's self-report. Ethics committee of the participating institution (Federal University of Bahia) approved the study (protocol number 694/2004) and informed consent was obtained from all participants.

For all three groups, we collected socio-demographic data such as age, sex, educational level and work status. The mBI is a 50-point scale that was applied to quantify impairment in activities of daily living such as grooming, walking, transferring, hygiene and voiding (50 points meaning completely independent for all activities) [9]. The NIHSS is a scale used to quantify stroke severity, scored 0 to 42 points for items such as motor and sensory deficits, ataxia and language (zero meaning lack of a measurable neurological deficit) and was applied by a medical student certified in applying the scale [10]. For stroke patients we also collected data on cerebral hemisphere affected and time from stroke onset to study admission. All scales were applied on the same day.

The Euro-QoL - 5 dimensions (EQ-5D) scale was used for QOL assessment [11]. The EQ-5D evaluates five QOL domains (mobility, pain, self-care, anxiety/depression and usual activities), each with one normal (no complaint) level and two increasingly abnormal levels [11,12]. In order to derive a composite score, each domain was weighted using a modeling equation, with total scores varying from 0 (death) to 1 (perfect health)[12]. As a reference mark, a score above 0.86 is considered normal in populational studies and scores above 0.78 are normal for patients aged between 65 and 74 years [13]. For the purpose of analysis, we compared total scores, weighted scores for each domain, and the proportion of patients with any complaint on each domain.

For statistical analysis we used the Statistical Package for the Social Sciences (SPSS) version 11.0. ANOVA test was used for comparing continuous variables between groups, with Scheffè's test for post-hoc comparisons. Categorical variables were compared using the Chi-square test for the three comparison groups, with the plan of further pairwise Chi-square testing in case of significance on the global test. Pearson's correlation coefficient was used for correlations between each scale. A P-value of < 0.05 was considered statistically significant.

## Results

From July, 2005 to November, 2007, 196 patients were evaluated, encompassing 67 patients with stroke, 62 with CHF and 67 caregivers. Table 1 shows the socio-demographic data, with study groups well-balanced for age and gender, but not for educational level, which was higher in the caregiver group when compared to the other groups (p < 0.001), but similar between the stroke and CHF patients. The proportion of patients without formal employment was high in all three groups (70-80%), reflecting the low socio-economical conditions of the population being studied. Most stroke patients suffered mild deficits as measured by the NIH Stroke Scale (median of four, range zero to 17). Mean (+/-SD) time from stroke onset to study recruitment was 28 +/- 36 months, median 12 months. No correlation was found between QOL and time since the stroke event (r = 0.018, P = 0.891).

**Table 1:** Socio-demographic data from 67 patients with stroke, 62 with congestive heart failure (CHF) and 67 caregivers

Variables	Stroke (a)	CHF (b)	Caregivers (c)
Age (years), mean (SD)	59.3 (13.3)	59.1 (12.3)	54.3 (14.2)
ANOVA P-value (DF)			0.052 (194)
P-value*		a/b = 0.996 b/c = 0.126	a/c = 0.098
Male sex (%)	44.8	37.1	31.3
P-value**	0.274		
Years of education, mean (SD)	4.4 (3.4)	5.6 (4.1)	8.7 (4.7)
ANOVA P-value (DF)			< 0.001 (189)
P-value*		a/b = 0.287 b/c < 0.001	a/c < 0.001
Proportion employed (%)	21.3	24.2	31.3
P-value**	0.406		

*Post-hoc Scheffè test; **Chi-square test; SD = standard deviation; DF = degrees of freedom.

Table 2 shows the results of QOL and functional profile evaluations. All three groups showed low QOL scores when compared to populational studies (expected score above 0.78). Stroke patients showed significantly lower EQ-5D scores when compared to caregivers (0.52 vs 0.65, p = 0.049) and CHF patients (0.52 vs 0.69, P = 0.010). The results remained significant when adjusting for educational level. In contrast, no difference was observed in overall EQ-5D scores between the CHF and caregiver groups. The same occurred in mBI evaluations, showing a greater impairment in activities of daily living of stroke patients when compared to caregivers (43.6 vs 50.0, P < 0.001) and with CHF patients (43.6 vs 49.8, P < 0.001), but not between CHF and caregiver groups.

**Table 2:** EQ-5D and modified Barthel Index (mBI) scores between study groups

Groups	EQ-5D, mean (SD)	P-value	BI, mean (SD)	P-value
Stroke (a)	0.52 (0.36)		43.6 (7.1)	
CHF (b)	0.69 (0.28)	a/b = 0.010 b/c = 0.812	49.8 (1.0)	a/b < 0.001 b/c = 0.971
Caregivers (c)	0.65 (0.24)	a/c = 0.049	50.0 (0.0)	a/c < 0.001
ANOVA P-value (DF)	0.006 (190)		< 0.001 (178)	

**Figure 1:**
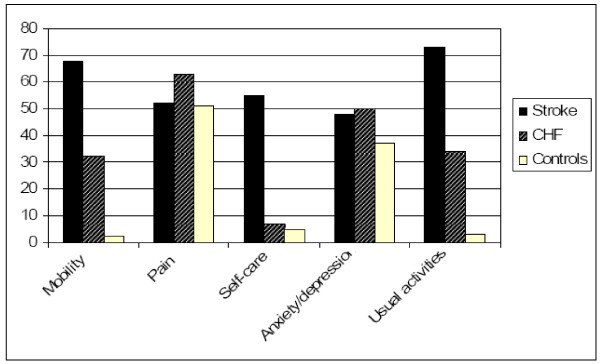
**Quality of life domains in the three comparison groups (stroke, congestive heart failure and caregivers)**. Proportion (%) of abnormal responses in EQ-5D domains of mobility, pain, self-care, anxiety/depression and usual activities between patients with stroke, congestive heart failure (CHF) and caregivers. Significant (p < 0.001) differences were noted in mobility, self-care and usual activity complaints. The only domain with a significant difference between stroke and caregivers, but not CHF and caregivers was self-care perception.

Weighted score results for each EQ-5D domain are shown in Table 3. Patients with stroke scored worse in QOL domains of mobility, self-care and usual activities when compared with CHF patients and the caregiver group (P < 0.001 for all comparisons, remaining significant after adjustment for educational level). CHF patients scored worse in domains of mobility and usual activities (P < 0.01 for all comparisons) but not in their perception of self-care. For the domains of pain and anxiety/depression there was no significant difference identified between the three groups. Similar results were observed when analyzing the proportion of patients with any complaint in each domain (Figure 1).

**Table 3:** Weighted score for each quality of life (QOL) domain in patients with stroke, congestive heart failure (CHF) and caregivers

QOL domains (EQ-5D)	Stroke (a)	CHF (b)	Caregivers (c)	ANOVA P-value (DF)	P-value
Mobility, mean (SD)	-0,05 (0,05)	-0,02 (0,03)	-0,001 (0,008)	< 0.001 (190)	a/b < 0,001 a/c < 0,001 b/c = 0,008
Dor Pain, mean (SD)	-0,10 (0,13)	-0,10 (0,11)	-0,07 (0,07)	0.088 (190)	a/b = 0,174 a/c = 0,998 b/c = 0,155
Self-care, mean (SD)	-0,08 (0,08)	-0,01 (0,03)	-0,00 (0,02)	< 0.001 (190)	a/b < 0,001 a/c < 0,001 b/c = 0,973
Anxiety/depression, mean (SD)	-0,07 (0,09)	-0,06 (0,08)	-0,03 (0,07)	0.052 (190)	a/b = 0,779 a/c = 0,061 b/c = 0,255
Usual activities, mean (SD)	-0,03 (0,03)	-0,01 (0,02)	-0,001 (0,01)	< 0.001 (190)	a/b < 0,001 a/c < 0,001 b/c = 0,003

DF = degrees of freedom.

The total EQ-5D score showed significant correlation with both mBI (r = 0.38, p < 0.001) and NIH Stroke Scale (r = -0.404, p = 0.001). No significant correlations were observed between total EQ-5D score and age or time from stroke onset. In patients with stroke, we observed a significantly worse deficit in right-hemisphere affected patients as compared with left-hemisphere: median NIH Stroke Scale score of six vs. three, p = 0.031; mean (+/-SD) mBI of 39+/-9 vs. 45+/-5, p = 0.041. Quality of life was slightly worse in right-hemisphere patients, but did not reach statistical significance (0.41+/-0.36 vs 0.59+/-0.36, p = 0.102).

## Discussion

In the present study, we demonstrated that stroke carries a significant impact in patient's perception of QOL. In other studies, EQ-5D scores were significantly lower (0.69 to 0.73) than caregivers, but higher than our stroke population (0.52)[14,15]. Stroke also carried a greater impact on QOL when compared to both CHF and caregiver groups. To our knowledge, only one other study compared different chronic diseases using the EQ-5D and showed that chronic cardiopathies carry a similar reduction in QOL as stroke and other chronic diseases, when compared to the general population [16]. However, the two studies differ considerably in regards to the population evaluated: in our study, the low educational level and high unemployment rate may have increased the impact of each disease in each individual's QOL. Comparing different chronic diseases in respect to their impact on QOL is relevant to health care organizations, both governmental and non-governmental, in regards to planning resource utilization.

When compared to the caregiver group, several QOL domains were affected in stroke patients. In previous studies, the domains most frequently affected were mobility, usual activities and self-care [17,18]. Most (> 50%) stroke patients in our study showed complaints in these same domains. Conversely, the CHF group demonstrated significant complaints in mobility and usual activities but no significant impact in self-care perception. Similarly, one previous study showed that CHF has an important impact on the ability of patients to perform their usual activities, with 76% of patients reporting problems in this dimension [16]. This indicated that patients felt that their disease made their recreational pastimes, sports or hobbies difficult, but fewer patients (24%) reported problems washing or dressing themselves [16].

This finding indicates that CHF patients still possess a feeling of independence despite significant impairment in daily activities. This contrasts to stroke patients, who despite a mild deficit (median NIHSS of four) still suffered a significant sense of dependence on caregivers. This differential impact of each disease in QOL domains is important, because health rehabilitation strategies should be tailored to each specific disease, such as including psychological support and occupational therapy for stroke patients to increase their sense of independence.

In regards to the anxiety/depression domain, previous studies show depression to be present in 30 to 40% of stroke patients [14,19-21], interfering with recovery, return to work and adherence to therapy. In one study, depression was the single most important determinant of QOL after in survivors up to one year after stroke onset [22]. In another study, depression was the most important determinant of motor deterioration in the second year after stroke onset [23]. Thus, it is not surprising in our study to find a high (almost 50%) prevalence of anxiety/depression complaints in stroke patients. However, the caregiver population also suffered a similar rate of complaints in this domain. This finding may be due to our caregiver population, composed of caregivers of stroke and CHF patients, who also suffer frequently of anxiety and depression [24-26].

Pain is a frequent complaint after stroke and has been shown to be significantly associated with a reduction in QOL [27]. However, in one study pain was found frequently (42%) but did not significantly affect QOL [28]. Similarly, our study shows pain as a frequent complaint in stroke patients, but not significantly different when compared to the caregiver or CHF groups.

Both stroke severity (measured by the NIHSS) and its impact on activities of daily living (measured by the mBI) correlated strongly with QOL. This finding was expected and was present despite a mild overall deficit measured by the NIHSS. Previous studies have also documented such a relationship [29,30]. Similar to our findings, others have documented significant reductions in QOL despite functional independence as measured in other scales [28,31], a fact that stresses the importance of measuring QOL as an outcome in stroke studies.

## Conclusions

The impact of stroke on individuals' quality of life is significantly greater in comparison to patients with congestive heart failure and caregivers. Patients with stroke, despite minor deficits, suffer from significant reduction of self-care perception.

## Competing interests

The authors declare that they have no competing interests.

## Authors' contributions

EBP conceived and carried out the study, and participated in the data analysis, drafting. IM participated in the acquisition of data for EQ-5D and mBI, and database management. JLBP participated in the acquisition of data for NIHSS and mBI, and database management. TGF, JCS, DFM, CC, ISN participated in the acquisition of data for NIHSS and mBI. PAPJ participated in the acquisition of data for NIHSS and stroke case definitions. JOF conceived and coordinated the study, participated in its design, stroke case definitions and statistical analysis. All authors read and approved the final manuscript.
